# Polygyny’s marriage squeeze is real but temporary

**DOI:** 10.1073/pnas.2605698123

**Published:** 2026-04-10

**Authors:** Jingyuan Deng

**Affiliations:** ^a^Department of Economics, University of Oxford, Oxford OX1 3UQ, United Kingdom

Gaddy et al. (1) make a valuable theoretical contribution by modeling how polygynous marriage markets can clear without producing permanent bachelors. However, their empirical claim that polygyny does not displace young men from the marriage market rests on measurement and specification choices that may attenuate estimates to the null.

## Measuring Polygyny at the Intensive Margin

The authors measure polygyny as the share of married men who are polygynous—the extensive margin only. Yet two wives per polygynous man produces a fundamentally different marriage squeeze than five. The ratio of currently married women to married men (the wife-husband ratio) captures both the extensive and intensive margins, as proposed by van de Walle (2) and employed by Jacoby (3).

This measure also avoids a systematic measurement problem. IPUMS identifies polygynous unions by linking women married to the same man within a census household. However, in sub-Saharan Africa, cowives frequently maintain separate dwellings within a homestead compound (4, 5). Census enumeration treating each dwelling as a separate household will systematically miss these unions. The wife-husband ratio sidesteps this problem entirely.

## Statistical Power

Running separate OLS regressions per census—often with fewer than 50 subnational localities—limits statistical power and attenuates results toward nullity. When the slope parameter is conceptually common across groups but intercepts differ, pooled estimation with group fixed effects is more statistically efficient (6).

## Reanalysis

**Fig. 1.**
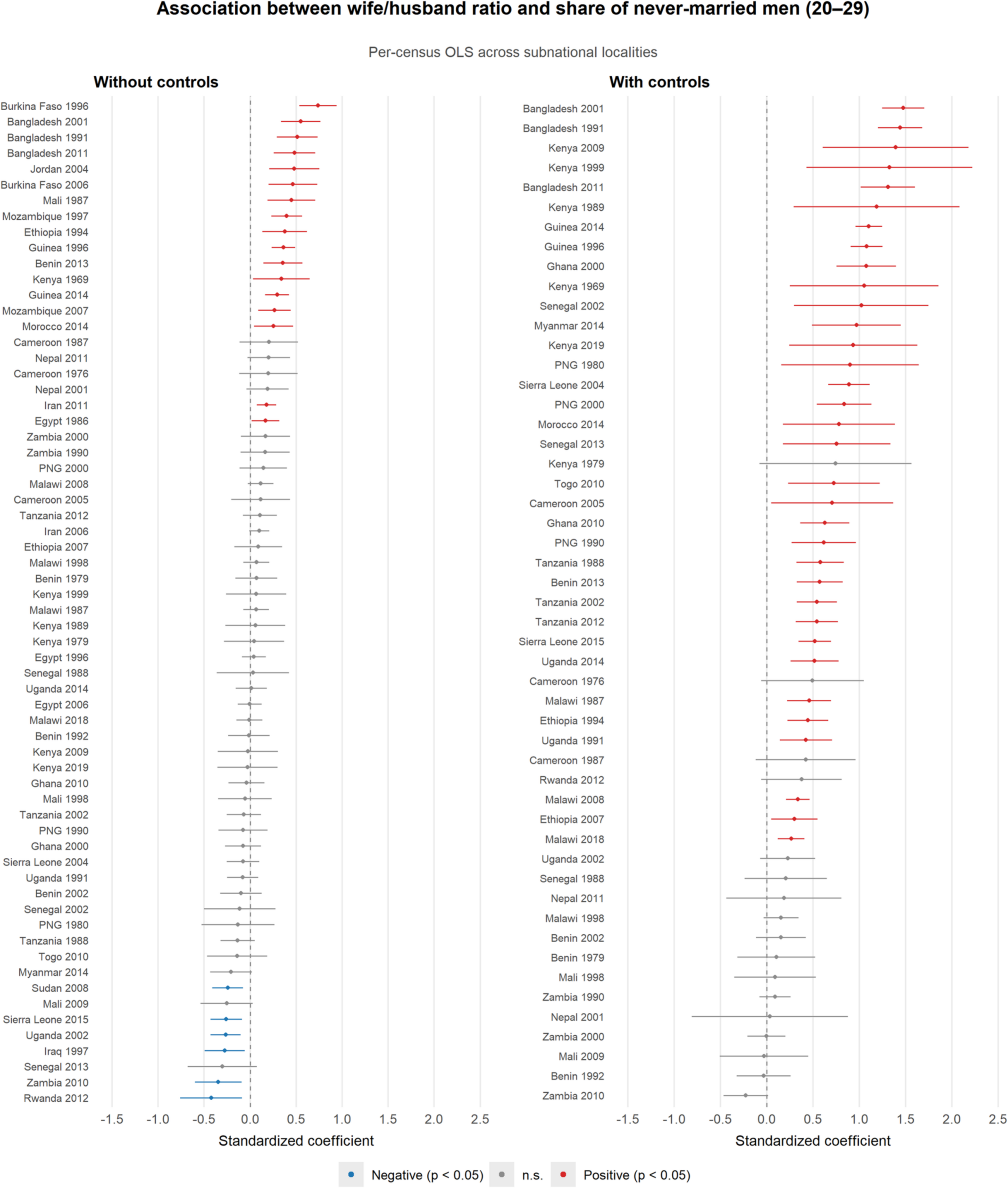
Per-census OLS coefficients of the wife-husband ratio on the share of never-married men aged 20 to 29, across 64 censuses in 27 countries. Each observation is a subnational locality in a census. *Left*: bivariate. *Right*: with controls (average education, log population density, sex ratio). Censuses missing coefficient plots on the right are due to unavailable data for the control variables. See Deng (7) https://zenodo.org/records/18732876 for replication data and code.

Using the same IPUMS International data across 58 censuses in 30 countries (due to unavailability of Nigeria), I replicate the figure 2 of Gaddy et al. (1)—OLS regressions of the share of never-married men aged 20 to 29 on polygyny intensity across subnational localities—replacing their extensive-margin measure with the wife-husband ratio. Fig. 1 presents per-census coefficients without and with locality-level controls (average years of schooling, log population density, and adult sex ratio). Most coefficients are positive, and controls strengthen the relationship, consistent with education proxying for marriage norms that confound the bivariate comparison.

**Fig. 2.**
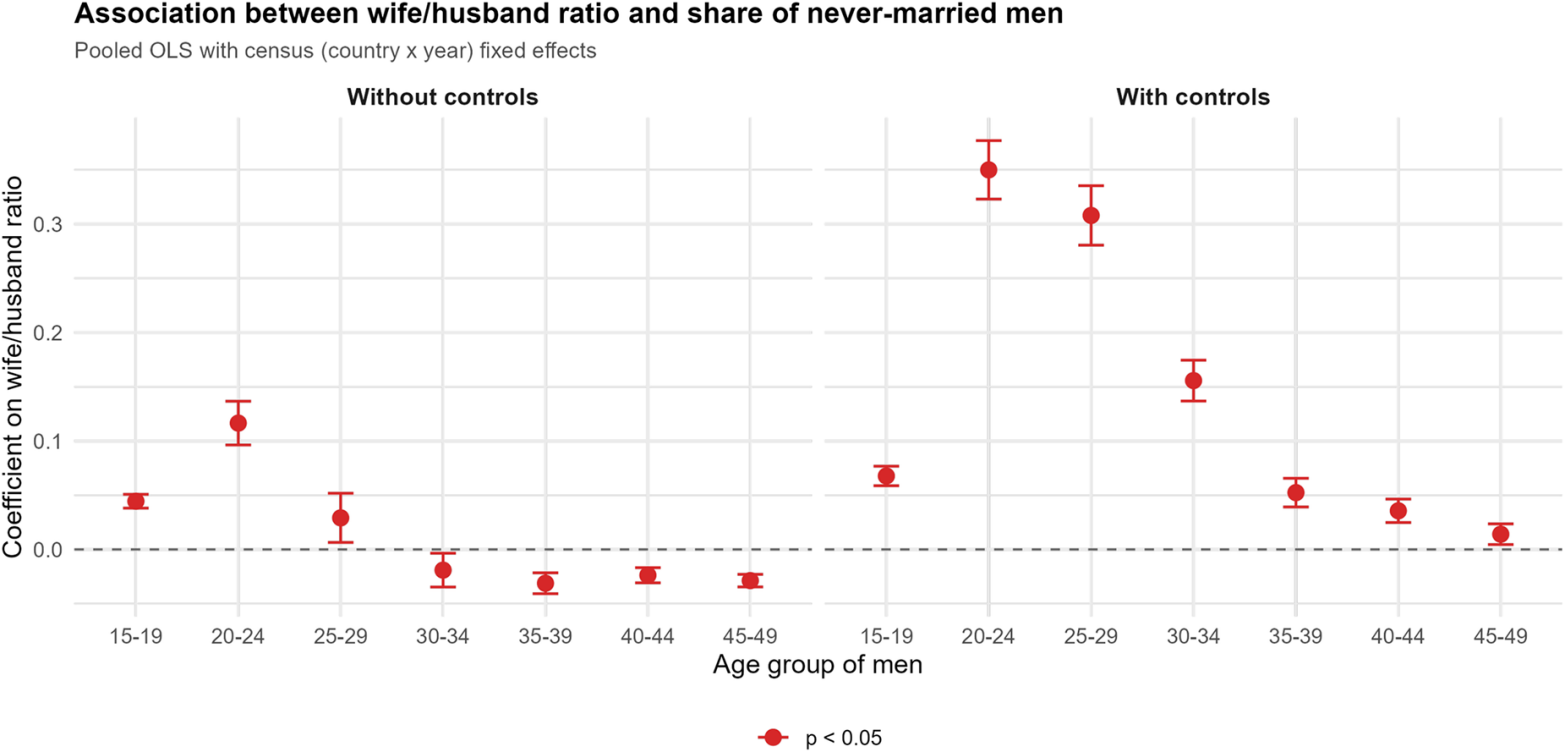
Pooled OLS coefficients of the wife-husband ratio on the share of never-married men by age group, with census (country × year) fixed effects. Each observation is a subnational locality in a census. *Left*: bivariate. *Right*: with controls. Censuses excluded due to unavailable data on the control variables in Fig. 1 are also excluded in this analysis. See Deng (7) https://zenodo.org/records/18732876 for replication data and code.

Fig. 2 presents pooled OLS estimates with census fixed effects across seven age bins from 15 to 19 to 45 to 49. The wife–husband ratio is a strong predictor of never-married status for men aged 15 to 29, with the coefficient peaking at ages 20 to 24 and attenuating monotonically to near zero by ages 40 to 49. Controls further sharpen this age gradient.

## Implications

The evidence suggests the truth lies between the authors’ conclusion and conventional wisdom. Polygyny does not produce permanent bachelors—as their model predicts—but it does delay marriage for young men. Moreover, male singlehood has an intensive margin: Years of delayed marriage carry social consequences independent of whether men eventually marry, and the true counterfactual marriage age absent polygyny may be lower still in more traditional societies. Since the literature linking polygyny to conflict, risk-taking, and instability operates primarily through unmarried young men, these mechanisms remain empirically well-supported.

## References

[1] H. Gaddy, R. Sear, L. Fortunato, High rates of polygyny do not lock large proportions of men out of the marriage market. Proc. Natl. Acad. Sci. U.S.A. **122**, e2508091122 (2025). 41042848 10.1073/pnas.2508091122 PMC12519187

[2] E. van de Walle , “Marriage in African censuses and inquiries” in *The Demography of Tropical Africa*, W. Brass, Ed. (Princeton University Press, Princeton, NJ, 1968).

[3] H. G. Jacoby, The economics of polygyny in Sub-Saharan Africa: Female productivity and the demand for wives in Côte d’Ivoire. J. Polit. Econ. **103**, 938–971 (1995).

[4] P. Draper, African marriage systems: Perspectives from evolutionary ecology. Ethol. Sociobiol. **10**, 145–169 (1989).

[5] B. B. Whiting, J. W. M. Whiting, Children of Six Cultures: A Psycho-Cultural Analysis (Harvard University Press, Cambridge, MA, 1975).

[6] J. M. Wooldridge, *Econometric Analysis of Cross Section and Panel Data* (MIT Press, Cambridge, MA, ed. 2, 2010).

[7] J. Deng, Replication data and code for “Polygyny’s marriage squeeze is real but temporary” (2026). Zenodo. https://zenodo.org/records/18732876.10.1073/pnas.260569812341961821

